# Effects of Gait Training with Lower-Limb Robotic Exoskeletons and Exoskeleton-Type Devices on Gait Symmetry and Gait Speed in Patients with Stroke: A Systematic Review and Meta-Analysis

**DOI:** 10.3390/bioengineering13080892

**Published:** 2026-08-02

**Authors:** Chengshuo Zhang, Jiarong Wu, Wanli Zang, Qiuxia Zhang

**Affiliations:** 1 School of Physical Education, Henan University of Science and Technology, Luoyang 471023, China; 2 School of Physical Education, Soochow University, Suzhou 215021, China

**Keywords:** stroke, lower-limb robotic exoskeleton, exoskeleton-type device, robot-assisted gait training, gait symmetry, gait speed, systematic review, meta-analysis

## Abstract

Gait asymmetry and reduced gait speed (GS) are common after stroke. This systematic review and meta-analysis evaluated the effects of gait training with lower-limb robotic exoskeletons or exoskeleton-type devices on gait asymmetry and GS compared with conventional rehabilitation or non-robotic gait training. PubMed, Embase, Web of Science, the Cochrane Library, and Scopus were searched from inception to 21 June 2026. Randomized controlled trials (RCTs) reporting spatial gait asymmetry (SGA), temporal gait asymmetry (TGA), or GS were included. Standardized mean differences (SMDs; Hedges’ g) and 95% confidence intervals (CIs) were pooled using random-effects models. Eleven RCTs involving 532 randomized participants were included. Training with these devices reduced SGA (SMD = −0.68, 95% CI −1.13 to −0.22, *p* < 0.01) and improved GS (SMD = 0.46, 95% CI 0.12 to 0.81, *p* = 0.01), but did not significantly affect TGA (SMD = −0.85, 95% CI −1.90 to 0.20, *p* = 0.11). The certainty of evidence, assessed using the Grading of Recommendations Assessment, Development and Evaluation (GRADE) framework, was very low for all three outcomes. Gait training with lower-limb robotic exoskeletons or exoskeleton-type devices may reduce SGA and improve GS after stroke, whereas its effect on TGA remains uncertain.

## 1. Introduction

Lower-limb motor control impairment after stroke can limit patients’ ability to stand and walk [1]. In a study of 487 patients with first-ever acute stroke, 44.1% had lower-limb motor impairment and 46.0% were unable to walk on admission [1]. Patients with some walking ability had an odds ratio of 9.48 for discharge home [1]. Gait abnormalities may persist even after patients regain community ambulation [2]. Among 54 community-ambulating patients with stroke, 55.5% had temporal gait asymmetry (TGA) and 33.3% had spatial gait asymmetry (SGA) [2]. The median self-selected gait speed (GS) in patients with chronic stroke is approximately 0.77 m/s [3]. Both GS and step-length asymmetry can affect the energy cost of walking [3]. These findings highlight the importance of improving gait symmetry and GS to support functional independence and community participation after stroke.

Rehabilitation training is an important component of stroke treatment and functional recovery [4]. Conventional rehabilitation aims to improve lower-limb muscle strength, motor coordination, and walking ability [4]. However, therapist-delivered gait training may be limited by therapist availability. It may also be difficult to provide a high volume of repetitive, task-specific walking practice during routine rehabilitation sessions [5,6]. Lower-limb robotic exoskeletons can provide repetitive and controllable movement assistance during walking practice. They can also guide the lower limbs through relatively consistent gait cycles [5,6,7,8]. These systems may support gait-pattern relearning by facilitating task-specific practice, affected-side loading, and bilateral coordination. However, their effects may vary according to device design, training mode, and patient characteristics [5,6,7,8].

Recent randomized controlled trials (RCTs) have evaluated several lower-limb robotic systems, including EXOWALK, LiteStepper, and curara® [9,10,11]. These trials have reported potential benefits for walking function, balance, gait parameters, or cortical activation. However, findings have varied across devices and outcomes, particularly for gait symmetry [9,10,11].

Previous reviews have mainly focused on walking independence, walking ability, balance, GS, or endurance, with limited attention to gait symmetry [7,8,12,13]. Few reviews have separately synthesized spatial and temporal gait asymmetry, although these outcomes may represent distinct dimensions of gait recovery. The present review therefore synthesized RCTs comparing gait training with lower-limb robotic exoskeletons or exoskeleton-type devices with conventional rehabilitation or non-robotic gait training. The effects on SGA, TGA, and GS were evaluated separately. The certainty of evidence was also assessed for each outcome. 

## 2. Materials and Methods

### 2.1. Protocol and Registration

This systematic review and meta-analysis was conducted in accordance with the Preferred Reporting Items for Systematic Reviews and Meta-Analyses 2020 (PRISMA 2020) statement [14]. The protocol was registered in the International Prospective Register of Systematic Reviews (registration number: CRD420261428795).

### 2.2. Information Sources and Search

We systematically searched PubMed, Embase, Web of Science, the Cochrane Library, and Scopus from database inception to 21 June 2026. The search strategy was developed according to the population, intervention, comparator, outcomes, and study design (PICOS) framework and adapted to the syntax of each database. The full search strategies are provided in Supplementary S1. In addition, we screened the reference lists of relevant systematic reviews, meta-analyses, and included studies to identify additional eligible studies.

### 2.3. Eligibility Criteria

Studies were selected according to the predefined PICOS framework. Trials meeting all of the following criteria were included in this meta-analysis: (1) Study design: RCTs evaluating the effects of gait training with lower-limb robotic exoskeletons or exoskeleton-type devices on gait symmetry or GS in patients with stroke. (2) Participants: patients aged ≥18 years with a diagnosis of ischemic or hemorrhagic stroke and lower-limb motor dysfunction, walking impairment, or hemiparetic gait. No restrictions were imposed on stroke stage or time since stroke. (3) Intervention and comparator: the intervention group received gait training assisted by a lower-limb robotic exoskeleton or exoskeleton-type gait training device, delivered either alone or in combination with conventional rehabilitation therapy. Training modes included overground gait training or treadmill gait training. Eligible robotic systems were classified as independently wearable lower-limb exoskeletons, mobile-frame exoskeleton-type systems, or platform-coupled exoskeleton-type systems. Independently wearable systems were defined as devices in which the joint-assisting components were worn directly on the lower limbs without dependence on an external robotic frame. Mobile-frame systems were eligible when limb-mounted or joint-guiding exoskeletal components were integrated with a movable body-support frame. Platform-coupled systems were eligible only when a limb-mounted orthotic or exoskeletal component was mechanically attached to the affected lower limb and directly provided joint-level assistance or gait-cycle guidance during walking. Conventional end-effector robots and treadmill systems without a limb-mounted or joint-guiding exoskeletal component were excluded. The control group received conventional rehabilitation therapy, conventional gait training, therapist-assisted walking training, conventional treadmill training, or other non-robotic-assisted walking training. (4) Outcomes: studies reported at least one of the following outcomes: SGA, TGA, or GS. Relevant measures included step length asymmetry, step length symmetry, spatial symmetry ratio or spatial step symmetry ratio, temporal symmetry ratio, swing-time asymmetry, temporal asymmetry, and GS. (5) Data requirements: studies were required to report group sample sizes and post-intervention means and standard deviations, or to provide sufficient information to calculate effect sizes from standard errors, 95% confidence intervals (CIs), *p* values, or other relevant statistics.

Studies were excluded if they met any of the following criteria: (1) non-randomized controlled trials; (2) participants were not patients with stroke; (3) interventions did not involve a lower-limb robotic exoskeleton or exoskeleton-type gait training device; (4) no eligible control group was included; (5) none of the outcomes of interest, namely SGA, TGA, or GS, was reported; (6) complete data required for meta-analysis could not be obtained or converted; (7) duplicate publications; or (8) reviews, conference abstracts, study protocols, case reports, or animal studies.

### 2.4. Data Collection and Extraction

The retrieved records were imported into EndNote X9 (Clarivate Analytics, Philadelphia, PA, USA) for reference management, and duplicate records were removed. Two reviewers (C.Z. and W.Z.) independently screened the titles and abstracts. They then assessed the full texts against the predefined eligibility criteria. The reviewers independently extracted the first author, publication year, study design, and country of each study. Participant characteristics included age, sample size, time since stroke, and baseline walking ability when reported. Outcome data included SGA, TGA, and GS. Device characteristics included the device name and its classification as independently wearable, mobile-frame, or platform-coupled. The reviewers also extracted the assisted side and joints, mechanical and body-support configuration, and assistance or control strategy. Training and comparator characteristics included required patient effort, therapist involvement, training mode and environment, training frequency, session duration, total number of sessions, intervention duration, concomitant rehabilitation, and control intervention. For quantitative synthesis, the sample sizes of the intervention and control groups were extracted. Baseline and post-intervention means and standard deviations were also extracted when available. Baseline data were used only to describe participant characteristics and assess between-group comparability. They were not used to calculate the pooled effect estimates. Because change scores and their corresponding standard deviations were inconsistently reported, all meta-analyses were based on post-intervention group means, standard deviations, and sample sizes. For studies reporting multiple assessment time points, data from the earliest assessment after completion of the intervention were used. Long-term follow-up data were not included in the primary analysis. When a study reported multiple GS measures, the primary GS outcome specified in the original trial was selected. If no primary outcome was specified, self-selected or comfortable GS was selected. When multiple measures were reported within the same gait-symmetry domain, the trial-defined primary outcome was selected. If no primary outcome was specified, the measure most comparable in definition and assessment method to those used in the other included studies was chosen. Only one measure per study was included in each pooled outcome to avoid double-counting. When means or standard deviations were not reported directly, they were derived from standard errors, 95% CIs, *p* values, or other available statistics when possible. Studies without sufficient data for a particular outcome were excluded only from the quantitative synthesis of that outcome. Disagreements between the two reviewers were resolved through discussion and, when necessary, adjudicated by a third reviewer (Q.Z.).

### 2.5. Risk-of-Bias Assessment

Two reviewers (C.Z. and W.Z.) independently assessed the risk of bias in each included RCT using the Cochrane risk-of-bias tool for randomized trials, version 2 (RoB 2) [15]. The assessment covered five domains: bias arising from the randomization process, bias due to deviations from intended interventions, bias due to missing outcome data, bias in measurement of the outcome, and bias in selection of the reported result. For each trial, domain-level and overall risk-of-bias judgments were classified as “low risk of bias,” “some concerns,” or “high risk of bias” according to the RoB 2 algorithm. Disagreements were resolved through discussion and, when necessary, adjudicated by a third reviewer (Q.Z.).

### 2.6. Data Synthesis and Analysis

Primary meta-analyses were performed using Stata 17.0 (StataCorp LLC, College Station, TX, USA), and the Pustejovsky–Rodgers test was conducted using R version 4.6.0 (R Foundation for Statistical Computing, Vienna, Austria) with the meta package, version 8.5.0. SGA, TGA, and GS were analyzed as continuous outcomes. Because outcome definitions and measurement methods varied across studies, post-intervention between-group effects were pooled as standardized mean differences (SMDs; Hedges’ g) with 95% CIs using the means, standard deviations, and sample sizes of the intervention and control groups. Effect directions were harmonized before pooling: measures for which higher values indicated better gait symmetry were reverse-coded so that negative SMDs consistently indicated reduced SGA or TGA, whereas positive SMDs indicated higher GS.

Between-study heterogeneity was assessed using Cochran’s Q test and the I^2^ statistic and interpreted alongside the direction of effects and the clinical characteristics of the included studies [16]. A *p* value < 0.10 for Cochran’s Q test was considered indicative of statistical heterogeneity, and I^2^ values of approximately 25%, 50%, and 75% were interpreted as low, moderate, and high heterogeneity, respectively [16]. Given the anticipated clinical variation in device classification, training mode, time since stroke, and control interventions, random-effects models were used for all pooled analyses. Between-study variance (τ^2^) was estimated using restricted maximum likelihood (REML) [17]. Potential sources of clinical heterogeneity, including age, time since stroke, baseline walking ability, device classification and technical characteristics, training mode and dose, concomitant rehabilitation, and control interventions, were extracted when reported and summarized descriptively. Subgroup analyses and meta-regression were considered when a sufficient number of studies and adequately reported study-level variables were available. Leave-one-out sensitivity analyses were performed to evaluate the influence of individual studies on the pooled effect estimates. Device-class sensitivity analyses were conducted by repeating the meta-analyses after restricting the included studies to independently wearable lower-limb exoskeletons. These analyses were used to examine whether the inclusion of mobile-frame or platform-coupled exoskeleton-type systems materially affected the pooled estimates. For outcomes with at least 10 studies, funnel plots were used to explore small-study effects and potential publication bias [18]. Because conventional Egger regression may yield artefactual associations when SMDs are correlated with their standard errors, funnel-plot asymmetry for outcomes meeting this criterion was assessed using the Pustejovsky–Rodgers test [19]. A two-sided *p* value < 0.10 was prespecified as evidence of small-study effects. For pooled effect estimates, a two-sided *p* value < 0.05 was considered statistically significant.

### 2.7. Certainty of Evidence

Two reviewers (C.Z. and W.Z.) independently assessed the certainty of evidence for each outcome using the Grading of Recommendations Assessment, Development and Evaluation (GRADE) framework [20]. Because no validated minimally important difference was available for either SGA or TGA, and the pooled GS effect could not be expressed in m/s, absolute SMDs of 0.20 and 0.50 were used as the primary and sensitivity thresholds, respectively. The corresponding optimal information sizes were approximately 800 and 128 participants, based on conventional assumptions (two-sided α = 0.05, 80% power, and equal group allocation). Imprecision was judged according to whether the optimal information size was met and whether the 95% confidence interval crossed the prespecified thresholds. Disagreements were resolved through discussion and, when necessary, adjudicated by a third reviewer (Q.Z.). Evidence from RCTs was initially rated as high certainty and assessed across five domains: risk of bias, inconsistency, indirectness, imprecision, and publication bias. Certainty was downgraded by one or two levels for serious or very serious concerns, respectively.

## 3. Results

### 3.1. Study Selection and Characteristics

**Figure 1:**
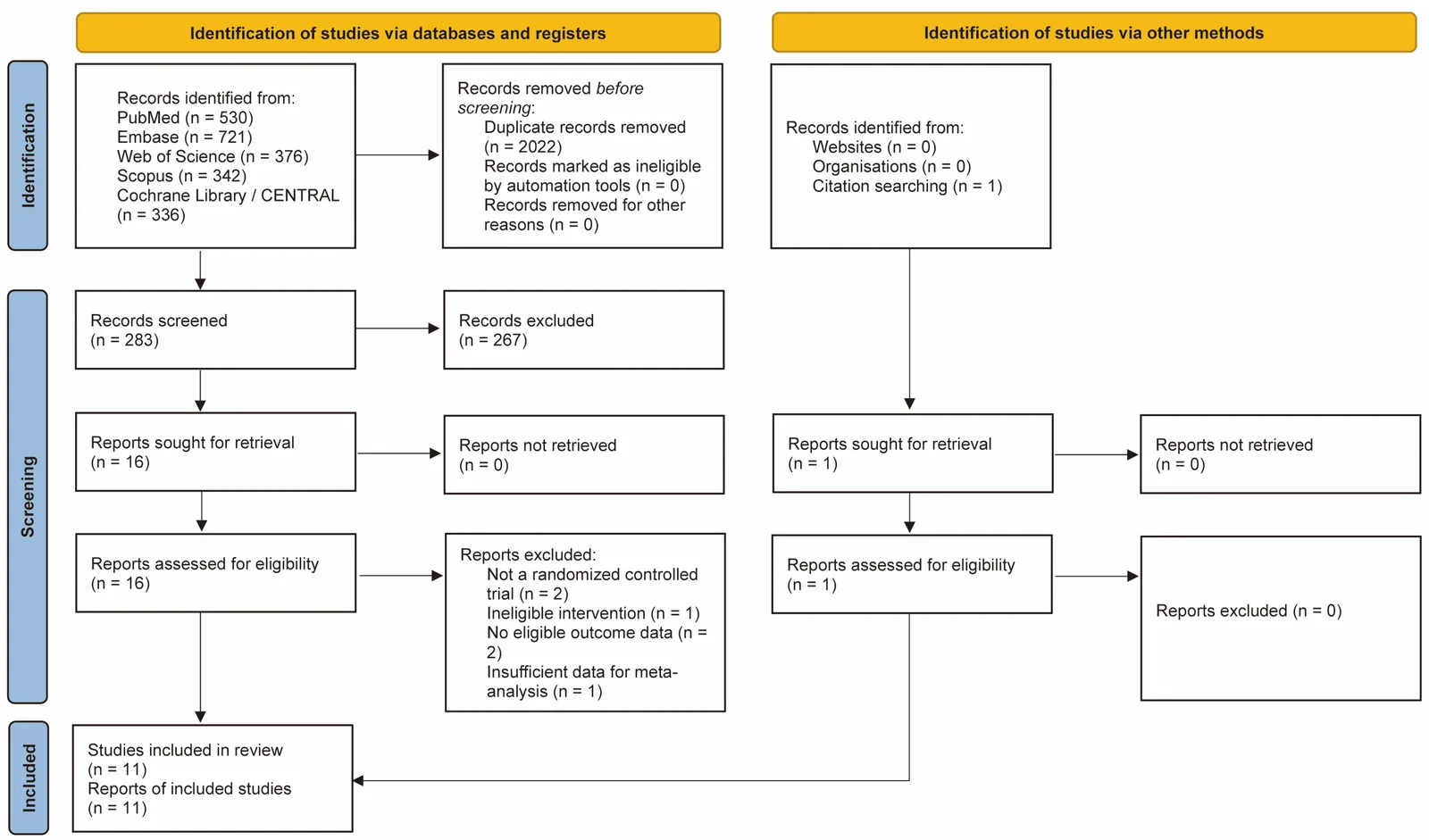
PRISMA 2020 flow diagram of the literature search and study selection process.

A total of 2305 records were identified through searches of five electronic databases, including PubMed, Embase, Web of Science, Scopus, and the Cochrane Library, and one additional Chinese-language record was identified through citation tracking. After removing 2022 duplicate records, 283 records underwent title and abstract screening. Of these, 267 records were excluded, and 16 articles proceeded to full-text assessment. Together with the additional article identified through citation tracking, 17 full-text articles were assessed for eligibility. After excluding six articles that did not meet the inclusion criteria, 11 RCTs were ultimately included in the meta-analysis. The study selection process is shown in Figure 1.

The 11 RCTs were published between 2019 and 2026 and included 532 randomized participants with stroke, with 266 participants assigned to the intervention groups and 266 assigned to the control groups. The trials were conducted in South Korea [9,21,22,23], China [10,24,25,26,27], Thailand [28], and Japan [11]. The intervention groups received lower-limb robotic exoskeleton-assisted or exoskeleton-type gait training, either alone or in combination with conventional rehabilitation therapy or conventional gait training. The evaluated devices included a robot for overground walking training [22], a platform-based robotic training device [28], EXOWALK [9], Kickstart [24,26], Healbot G [23], a soft exoskeleton [25], SUBAR [21], BEAR-H1 [27], LiteStepper® [10], and curara® [11]. Based on their mechanical configuration, eight trials evaluated independently wearable systems [10,11,22,23,24,25,26,27], two evaluated mobile-frame systems, namely EXOWALK and SUBAR [9,21], and one evaluated the platform-coupled Welwalk system [28]. Detailed device classifications and technical characteristics are presented in Supplementary Table S2, and the corresponding training protocols are presented in Supplementary Table S1. The Welwalk system used by Thimabut et al. [28] was included because a knee–ankle–foot robotic orthosis was attached directly to the paretic lower limb and provided gait-cycle-dependent knee assistance during treadmill walking. It was therefore classified as a platform-coupled exoskeleton-type system rather than a conventional end-effector robot or treadmill-only system. EXOWALK [9] and SUBAR [21] were classified as mobile-frame exoskeleton-type systems because their exoskeletal or joint-guiding components were integrated with movable external body-support frames. The control groups mainly received conventional rehabilitation therapy, conventional walking training, therapist-assisted gait training, conventional treadmill gait training, conventional assisted walking training, or conventional physical therapy [9,10,11,21,22,23,24,25,26,27,28]. The time since stroke ranged from approximately 0.6 to 189.6 months, and one trial did not report this information [11]. The intervention duration ranged from 15 days to 6 weeks, with variation in training frequency, session duration, total number of sessions, and training mode. Five trials contributing to the SGA and TGA meta-analyses randomized 314 participants [9,22,23,25,26]. Post-intervention data for each outcome were available for 196 participants, reflecting unavailable or incomplete outcome-specific gait-assessment data. All 11 trials contributed to the GS analysis, comprising 469 analyzed participants; six trials contributed only to the GS meta-analysis [10,11,21,24,27,28]. Study-specific randomized and analyzed sample sizes and the reported reasons for unavailable data are presented in Supplementary Table S3. The basic characteristics of the included studies are summarized in Table 1.

### 3.2. Risk-of-Bias Results

**Figure 2:**
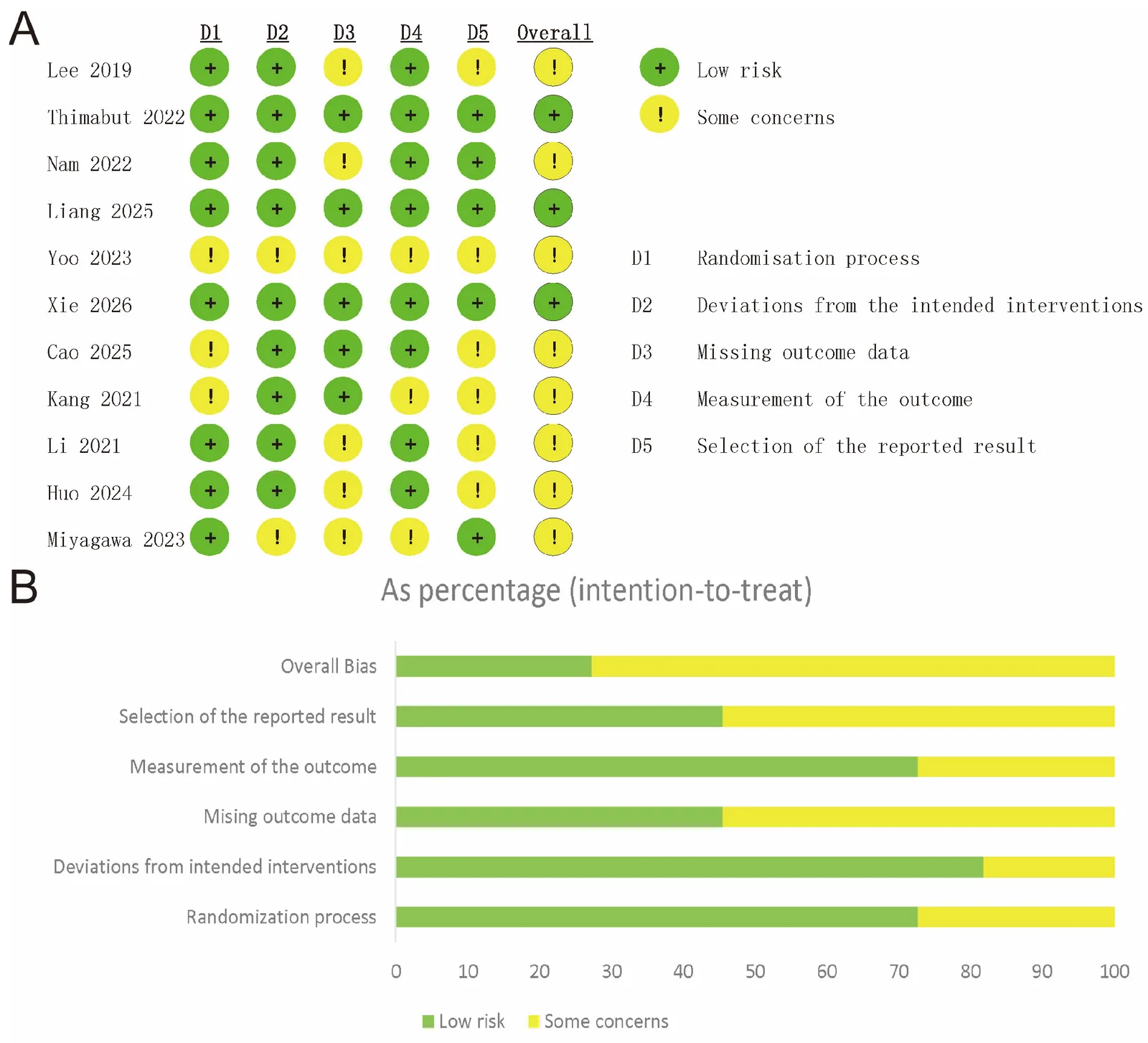
Risk-of-bias assessments for all 11 included trials using the Cochrane risk-of-bias tool for randomized trials, version 2 (RoB 2) [9,10,11,21,22,23,24,25,26,27,28]: (**A**) traffic-light plot of domain-level and overall judgments; and (**B**) summary plot showing the percentage distribution of judgments across risk-of-bias domains.

The RoB 2 assessments for the 11 included trials are summarized in Figure 2. Overall, three trials were judged to have a low risk of bias and eight were judged to have some concerns; none was judged to have a high risk of bias. The main concerns related to missing outcome data and selection of the reported result, although concerns were also identified in some trials regarding the randomization process, deviations from intended interventions, and measurement of the outcome.

### 3.3. Results for SGA

**Figure 3:**
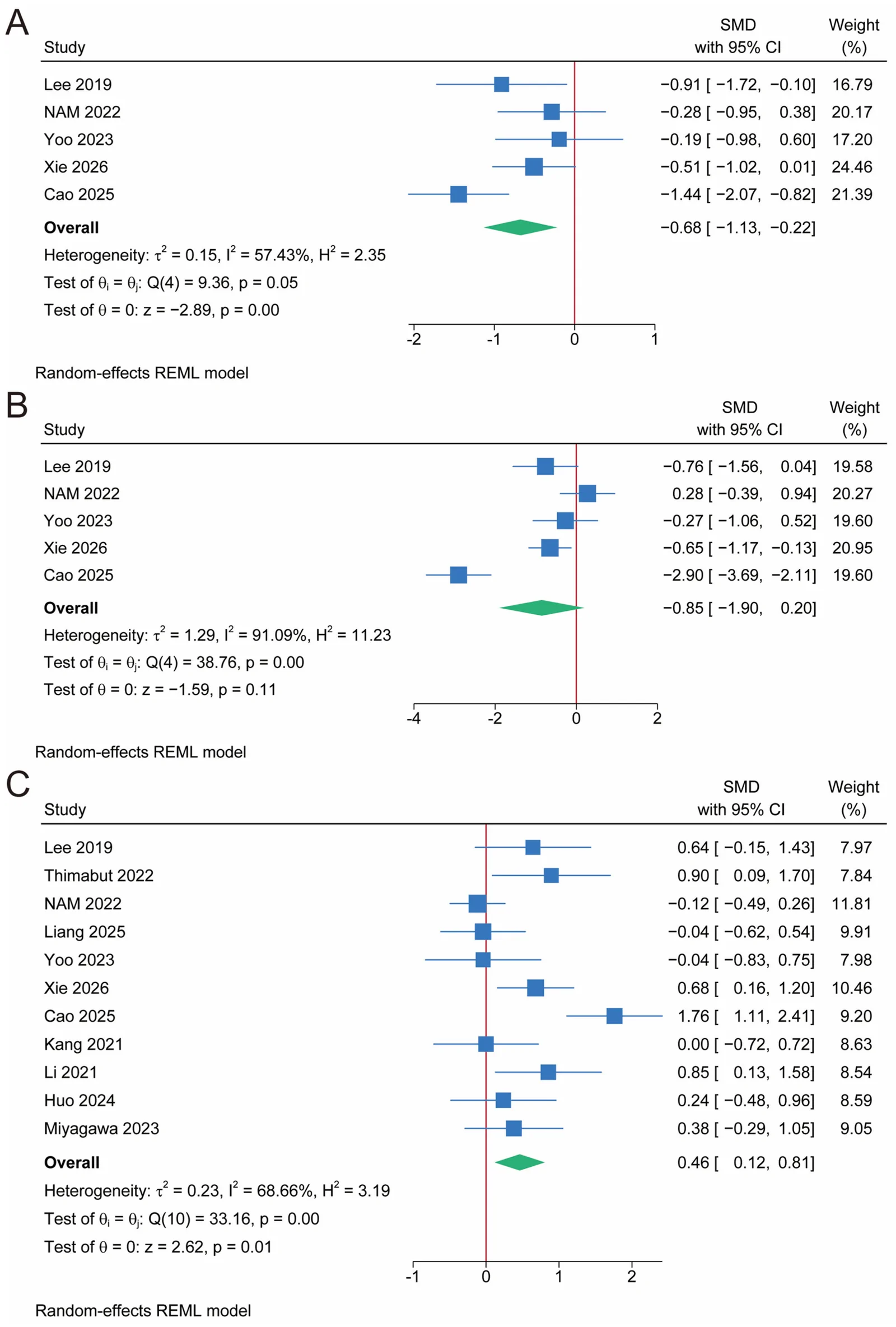
Forest plots of the meta-analysis of the effects of gait training with lower-limb robotic exoskeletons or exoskeleton-type devices on gait symmetry and gait speed in patients with stroke: (**A**) spatial gait asymmetry (SGA) [9,22,23,25,26]; (**B**) temporal gait asymmetry (TGA) [9,22,23,25,26]; and (**C**) gait speed [9,10,11,21,22,23,24,25,26,27,28]. Squares represent study-specific effect estimates, horizontal lines represent 95% confidence intervals, and diamonds represent pooled effect estimates. SMD, standardized mean difference; CI, confidence interval; REML, restricted maximum likelihood.

This meta-analysis included five studies involving 196 patients with stroke who provided post-intervention SGA data [9,22,23,25,26]. Given the clinical differences among the included studies in intervention device type, training mode, and time since stroke, a random-effects model was used for the pooled analysis. The results showed that SGA was reduced in the intervention group compared with the control group, with a statistically significant between-group difference (SMD = −0.68, 95% CI −1.13 to −0.22, *p* < 0.01) (Figure 3A). Moderate heterogeneity was observed across studies (I^2^ = 57.43%, *p* = 0.05).

### 3.4. Results for TGA

This meta-analysis included five studies involving 196 patients with stroke that reported post-intervention TGA [9,22,23,25,26]. Given the clinical differences among the included studies in intervention device type, training mode, and time since stroke, a random-effects model was used for the pooled analysis. The results showed no statistically significant between-group difference in TGA between the intervention and control groups (SMD = −0.85, 95% CI −1.90 to 0.20, *p* = 0.11) (Figure 3B). High heterogeneity was observed across studies (I^2^ = 91.09%, *p* < 0.01).

### 3.5. Results for GS

**Figure 4:**
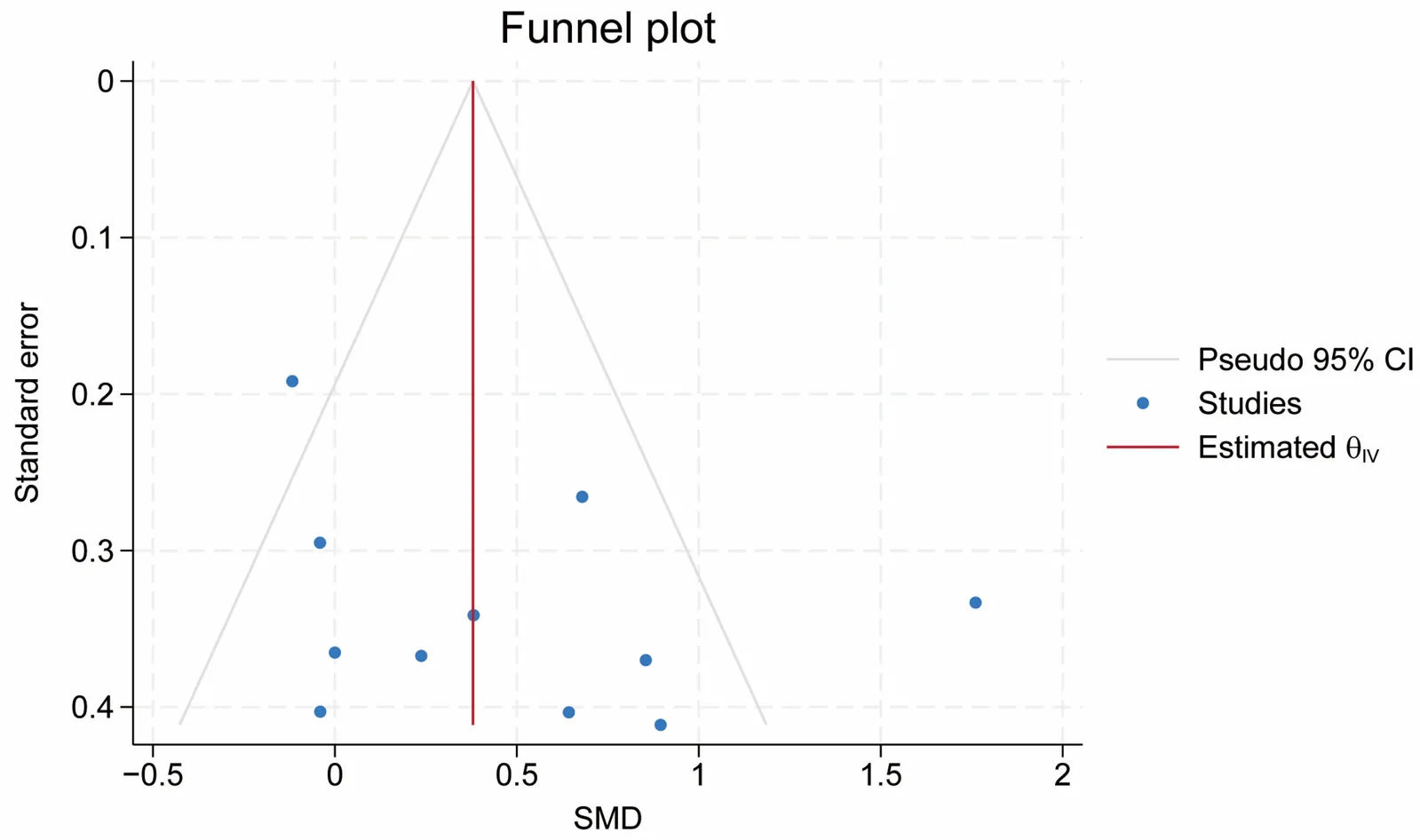
Funnel plot of the effect of gait training with lower-limb robotic exoskeletons or exoskeleton-type devices on gait speed in patients with stroke. SMD, standardized mean difference; CI, confidence interval.

This meta-analysis included 11 studies involving 469 patients with stroke who provided post-intervention GS data [9,10,11,21,22,23,24,25,26,27,28]. Given the clinical differences among the included studies in intervention device type, training mode, and time since stroke, a random-effects model was used for the pooled analysis. The results showed that post-intervention GS was higher in the intervention group than in the control group, with a statistically significant between-group difference (SMD = 0.46, 95% CI 0.12 to 0.81, *p* = 0.01) (Figure 3C). Moderate heterogeneity was observed across studies (I^2^ = 68.66%, *p* < 0.01). The funnel plot for GS was visually asymmetric, with several studies deviating to the right. However, the Pustejovsky–Rodgers test did not indicate statistically significant small-study effects (t = 0.456, degrees of freedom = 9, *p* = 0.6594). Therefore, the visual asymmetry was interpreted cautiously and was not considered sufficient evidence of publication bias (Figure 4).

### 3.6. Sensitivity Analysis

Leave-one-out sensitivity analyses were performed to assess the influence of individual studies on the stability of the pooled effect estimates. For SGA, after sequentially excluding each study, the pooled effect estimates ranged from SMD = −0.78 to −0.46, and all results remained statistically significant (*p* = 0.003–0.029), suggesting that the pooled result for SGA was generally stable. For TGA, after sequentially excluding each study, the pooled effect estimates ranged from SMD = −1.14 to −0.36, and none reached statistical significance (*p* = 0.053–0.204), indicating that the non-significant finding for TGA generally remained unchanged. However, this outcome should still be interpreted with caution because of the relatively large fluctuations in effect estimates and wide confidence intervals. For GS, after sequentially excluding each study, the pooled effect estimates ranged from SMD = 0.31 to 0.54, and all results remained statistically significant (*p* = 0.003–0.024), suggesting that the pooled result for GS was relatively robust. The leave-one-out sensitivity analyses are shown in Supplementary Figure S1.

In the device-class sensitivity analyses, exclusion of the mobile-frame EXOWALK trial [9] resulted in a statistically significant reduction in SGA (SMD = −0.77, 95% CI −1.31 to −0.24, *p* < 0.01; I^2^ = 60.76%), whereas the pooled effect on TGA remained non-significant (SMD = −1.14, 95% CI −2.29 to 0.01, *p* = 0.053; I^2^ = 90.39%). After excluding the platform-coupled Welwalk trial and the mobile-frame EXOWALK and SUBAR trials [9,21,28], the pooled effect on GS remained statistically significant (SMD = 0.56, 95% CI 0.15 to 0.98, *p* = 0.01; I^2^ = 67.01%). Therefore, excluding systems not classified as independently wearable exoskeletons did not materially change the direction or statistical significance of the primary findings. The device-class sensitivity analyses are shown in Supplementary Figure S2.

### 3.7. GRADE Certainty of Evidence Assessment

The GRADE certainty-of-evidence assessment is summarized in Table 2. Certainty was downgraded by one level for risk of bias and imprecision for all three outcomes. For inconsistency, certainty was downgraded by one level for SGA and GS and by two levels for TGA; no downgrading was applied for indirectness. Publication bias was not assessed for SGA or TGA because fewer than 10 studies contributed to each analysis and was not downgraded for GS. Overall, the certainty of evidence was rated as very low for SGA, TGA, and GS.

## 4. Discussion

This meta-analysis of 11 RCTs involving 532 randomized participants with stroke found that gait training with lower-limb robotic exoskeletons or exoskeleton-type devices may reduce SGA and improve GS, whereas its effect on TGA remains uncertain. Sensitivity analyses supported the robustness of the SGA and GS findings but showed considerable instability for TGA. Given the very low certainty of evidence for all three outcomes, these findings should be interpreted cautiously.

The present findings are partly consistent with previous evidence. The improvement in GS is consistent with previous systematic reviews and meta-analyses reporting beneficial effects of robot-assisted gait training on walking speed and walking ability [7,8,12,13,29]. The reduction in SGA extends the previous evidence because earlier reviews rarely analyzed spatial and temporal gait asymmetry separately. In contrast, the non-significant result for TGA should not be considered directly contradictory to previous reports of improved walking ability, balance, or GS, because temporal symmetry represents a distinct dimension of gait recovery and may not improve in parallel with overall walking performance. Differences in stroke stage, baseline walking ability, robotic device design, training dose, control interventions, and outcome definitions may partly explain the variation among studies. Training dose is also an important determinant of motor recovery after stroke [30].

Gait symmetry reflects coordination between the affected and unaffected lower limbs during the gait cycle in patients with stroke [2,3]. SGA mainly captures spatial differences, such as bilateral step length, whereas TGA reflects temporal differences, such as swing time and stance time [2,31]. In this meta-analysis, training with these devices was associated with a significant reduction in SGA. This effect may be partly explained by their ability to provide movement assistance, guide a more standardized gait cycle, promote affected-side weight-bearing, and increase the number of effective gait cycles [5,7,8], thereby supporting bilateral step-length coordination [31,32]. However, spatial gait symmetry measures and calculation methods were not fully consistent across studies [9,22,23,25,26,33], and no unified minimal clinically important difference was identified for interpreting the pooled SGA effect. Therefore, although the SGA improvement was statistically significant, its clinical importance and direct relevance to daily walking function remain uncertain.

GS is closely related to functional independence and community mobility after stroke, and higher walking speed generally indicates better daily walking capacity [34]. In this meta-analysis, training with these devices was associated with improved GS. This improvement may be partly explained by the ability of robotic exoskeletons to provide repetitive and relatively stable movement assistance, increase the number of effective gait cycles within a limited training period, and reduce abnormal compensatory patterns [5,7,8,29,35]. The direction of this finding is broadly consistent with previous meta-analyses, which reported beneficial effects of wearable or overground exoskeleton training on GS and walking ability, although the magnitude of benefit and certainty of evidence varied across reviews [7,8,29]. However, because this study used SMDs to pool GS outcomes measured with different methods and units, the pooled effect cannot be directly converted into m/s or used to determine whether the minimal clinically important difference for GS was reached [36]. In addition, moderate heterogeneity and visual funnel-plot asymmetry warrant caution. However, the Pustejovsky–Rodgers test did not detect statistically significant small-study effects (*p* = 0.6594), and publication bias was therefore not downgraded in the GRADE assessment. The potential physiological effects of training with these devices may be mediated through repetitive, task-specific sensorimotor practice. By providing controlled lower-limb assistance and repeated gait-cycle guidance, these systems may increase affected-side loading, enhance proprioceptive and somatosensory feedback, and promote more coordinated bilateral muscle activation. Repeated sensory input and motor practice may facilitate use-dependent neuroplasticity and reorganization of motor cortical networks. Consistent with this interpretation, included trials using functional near-infrared spectroscopy reported increased or more balanced cortical activation after exoskeleton-assisted training [10,23]. These mechanisms may partly contribute to improvements in gait symmetry and gait speed; however, they were not directly evaluated in the present meta-analysis and should therefore be regarded as plausible explanations rather than confirmed causal mechanisms.

Unlike SGA and GS, the current evidence is insufficient to confirm whether training with these devices improves TGA. TGA involves temporal coordination between the affected and unaffected lower limbs during the swing, stance, and double-support phases [2,37], and may be influenced by balance control, sensory input, baseline motor function, and compensatory strategies [37,38]. Previous studies have suggested that SGA and TGA do not necessarily recover synchronously and may respond differently to the same training stimulus [39]. Patterson et al. found that improvements in GS, balance, and functional activities were more common than improvements in step-length or swing-time symmetry, with many patients continuing to show gait asymmetry despite gains in overall walking ability [39]. These findings suggest that GS, SGA, and TGA may reflect distinct dimensions of gait recovery, and that temporal gait parameters may not improve in parallel with overall walking ability. In the present review, differences in time since stroke, baseline walking ability, exoskeleton control strategies, training environments, and TGA calculation methods may have contributed to the high heterogeneity [9,22,23,25,26]. The sensitivity analysis further indicated that the TGA finding was unstable, supporting a cautious interpretation of this outcome.

Previous reviews mainly focused on walking independence, walking ability, balance, GS, or endurance, with limited distinction between spatial and temporal gait asymmetry [7,8,12,13]. In addition, previous studies were generally limited by small samples, heterogeneous robotic systems and training protocols, inconsistent gait-symmetry definitions, and short intervention or follow-up periods. The present study addresses an important part of this gap by separately pooling SGA, TGA, and GS, thereby distinguishing spatial coordination, temporal coordination, and overall walking performance. However, the available evidence remains insufficient to determine which device, training protocol, or patient subgroup is most likely to benefit. This study has several limitations. First, the time since stroke varied markedly across studies, which may have influenced spontaneous recovery, baseline walking ability, and responsiveness to gait training. Because the number of studies was limited and this variable was reported inconsistently, its influence could not be reliably explored through subgroup analysis or meta-regression. In addition, most participants were able to complete some degree of walking training and gait assessment; therefore, the findings may not be applicable to patients with severe lower-limb dysfunction or those who are completely unable to walk independently. Second, the included systems were mechanically heterogeneous. Independently wearable, mobile-frame, and platform-coupled devices differed in body-weight support, assisted joints, patient effort, therapist involvement, control strategy, and training environment. Training protocols also varied in frequency, intensity, session duration, total sessions, and intervention duration. Although device-class sensitivity analyses were consistent with the primary analyses, the small number of non-wearable trials precluded formal subgroup comparisons. Accordingly, the pooled estimates represent average effects across heterogeneous device classes and training regimens, and residual confounding cannot be excluded. Third, the number of studies was limited, particularly for SGA and TGA, and reliable subgroup analyses or meta-regression could not be performed to explore sources of heterogeneity. Fourth, the included trials did not systematically assess free-living walking behavior after training. Because gait symmetry and GS were primarily measured under structured clinical or laboratory conditions, it remains unclear whether the observed changes translated into greater walking frequency, duration, distance, or mobility across different real-world environments. Fifth, this study calculated effect sizes using post-intervention means and standard deviations rather than consistently using change scores or baseline-adjusted effect estimates. In some small-sample trials with baseline imbalances or insufficient baseline comparability, the pooled results may have been influenced by residual baseline differences. Finally, this study did not systematically search grey literature or clinical trial registries, and unpublished or ongoing studies may have been missed.

Regarding methodological quality, the RoB 2 assessment showed that most included studies had some concerns, mainly related to selection of the reported result and missing outcome data. Some studies also had limitations in randomization, outcome measurement, or deviations from intended interventions. These issues may reduce the credibility of the pooled estimates. Although blinding of patients and therapists is inherently difficult in robotic device-assisted rehabilitation trials, future studies should improve randomization and allocation concealment, implement blinded outcome assessment whenever possible, prospectively register protocols, and report outcome data completely. In addition, safety was not prespecified as an outcome in this review, and adverse events such as falls, pain, skin injury, and device-related events were reported inconsistently. Consistent with recommendations for transparent harms reporting in randomized trials [40], future studies should systematically define and report adverse events. Therefore, the current evidence does not allow a definitive judgment regarding the safety of training with these devices. Future multicenter RCTs should use standardized definitions and calculation methods for SGA and TGA, report device characteristics and training dose in detail, stratify participants according to stroke stage and baseline walking ability, extend follow-up duration, and systematically assess adherence, adverse events, neurophysiological changes, and the transfer of gait improvements to daily walking and social participation [41]. Future studies should also complement clinical gait assessments with wearable sensors to quantify free-living walking frequency, duration, and distance [42], and with context-aware monitoring frameworks to characterize the locations and environmental contexts in which walking occurs [43].

## 5. Conclusions

This meta-analysis suggests that gait training with lower-limb robotic exoskeletons or exoskeleton-type devices may reduce SGA and improve GS in patients with stroke, whereas its effect on TGA remains uncertain. The certainty of the evidence was very low for SGA, TGA, and GS; therefore, these findings should be interpreted with caution. Future adequately powered RCTs should standardize gait symmetry measures and training protocols, systematically record adverse events, and determine whether improvements in gait symmetry and GS translate into sustained gains in walking ability, independence in activities of daily living, and social participation across different stroke stages and functional levels.

## Figures and Tables

**Table 1:** Basic characteristics of the included studies.

Included Study	Country	Sample Size (C/T)	Age (C/T) Years	Time Since Stroke (C/T)	Intervention Group	Control Group	Intervention Duration	Outcomes
Lee et al. 2019 [22]	South Korea	14/14	62.2 ± 6.36/ 61.85 ± 7.87	50.46 ± 10.23 months/48.82 ± 8.68 months	CRT + conventional walking training + robot for overground walking training	CRT + conventional walking training	4 weeks	SGA, TGA, GS
Thimabut et al. 2022 [28]	Thailand	13/13	62.80 ± 8.50/ 52.80 ± 12.60	2.4 ± 0.7 months/ 1.8 ± 0.8 months	CRT + platform-based robotic training device	CRT + conventional walking training	6 weeks	GS
Nam et al. 2022 [9]	South Korea	72/72	62.42 ± 15.04/ 60.63 ± 15.61	17.2 ± 40.1 months/25.2 ± 47.2 months	CRT + EXOWALK exoskeleton-assisted gait training	CRT + therapist-assisted gait training	4 weeks	SGA, TGA, GS
Liang et al. 2025 [24]	China	23/23	61.83 ± 12.30 /60.43 ± 11.55	1.46 ± 0.95 months/1.31 ± 0.19 months	CRT + Kickstart^®^ lower-limb exoskeleton-assisted walking training	CRT + conventional walking training	4 weeks	GS
Yoo et al. 2023 [23]	South Korea	15/15	66.00 ± 5.31 /61.86 ± 7.10	189.6 ± 85.7 months/138.8 ± 89.9 months	Healbot G powered exoskeleton-assisted treadmill gait training	Conventional treadmill gait training	4 weeks	SGA, TGA, GS
Xie et al. 2026 [25]	China	30/30	61.80 ± 5.55/ 61.63 ± 5.90	1.92 ± 1.36 months/2.23 ± 1.88 months	CRT + bilateral soft exoskeleton-assisted treadmill gait training	CRT + conventional treadmill gait training	20 consecutive days	SGA, TGA, GS
Cao et al. 2025 [26]	China	26/26	51.75 ± 12.81 /52.08 ± 12.9	0.58 ± 0.14 months/0.55 ± 0.16 months	CRT + Kickstart wearable exoskeleton combined with posture-feedback walking training	CRT + conventional assisted walking training	4 weeks	SGA, TGA, GS
Kang et al. 2021 [21]	South Korea	15/15	62.9 ± 6.0 /64.3 ± 4.6	42.6 ± 59.2 months/168.3 ± 67.3 months	SUBAR overground lower-limb robotic exoskeleton-assisted gait training	Conventional physical therapy, including functional gait training	3 weeks	GS
Li et al. 2021 [27]	China	18/18	50.13 ± 9.49/50.53 ± 12.26	3.38 ± 1.19 months/2.53 ± 1.33 months	CRT + BEAR-H1 lower-limb robotic exoskeleton-assisted gait training	CRT + conventional walking training	4 weeks	GS
Huo et al. 2024 [10]	China	20/20	55.25 ± 11.16 /57.93 ± 11.47	2.43 ± 1.09 months/2.24 ± 1.17 months	CRT + LiteStepper^®^ unilateral lower-limb robotic exoskeleton-assisted overground gait training	CRT + conventional walking training	4 weeks	GS
Miyagawa et al. 2023 [11]	Japan	20/20	63.0 ± 12.9 /65.1 ± 12.9	Not reported	CRT + curara^®^ wearable powered robot-assisted gait training	CRT + therapist-assisted conventional gait training	15-day trial period	GS

Note: C = control group; T = intervention group; CRT = conventional rehabilitation therapy; SGA = spatial gait asymmetry; TGA = temporal gait asymmetry; GS = gait speed. Detailed device classifications and technical characteristics are presented in Supplementary Table S2. Sample sizes in Table 1 refer to randomized participants; outcome-specific analyzable sample sizes are presented in Supplementary Table S3.

**Table 2:** GRADE certainty of evidence for the effects of gait training with lower-limb robotic exoskeletons or exoskeleton-type devices on gait symmetry and gait speed in patients with stroke.

Outcome	Included Studies/Sample Size	Risk of Bias	Inconsistency	Indirectness	Imprecision	Publication Bias	Certainty of Evidence
SGA	5 studies/196	Serious	Serious	Not serious	Serious	Not assessed (number of studies < 10)	Very low
TGA	5 studies/196	Serious	Very serious	Not serious	Serious	Not assessed (number of studies < 10)	Very low
GS	11 studies/469	Serious	Serious	Not serious	Serious	Not serious	Very low

Note: SGA = spatial gait asymmetry; TGA = temporal gait asymmetry; GS = gait speed; GRADE = Grading of Recommendations Assessment, Development and Evaluation. Evidence from randomized controlled trials was initially rated as high certainty. Risk of bias was downgraded by one level for all outcomes because most contributing trials were judged to have some concerns—four of the five trials contributing to the SGA and TGA analyses and eight of the 11 trials contributing to the GS analysis; no trial was judged to have a high risk of bias. Inconsistency was downgraded by one level for SGA (I^2^ = 57.43%) and GS (I^2^ = 68.66%) and by two levels for TGA (I^2^ = 91.09%). No downgrading was applied for indirectness. Imprecision was downgraded by one level for all outcomes because none met the optimal information size of approximately 800 participants for a standardized mean difference threshold of 0.20 and each confidence interval crossed at least one prespecified effect threshold, although all outcomes exceeded the corresponding optimal information size of approximately 128 participants for a threshold of 0.50. Publication bias was not assessed for SGA or TGA because fewer than 10 studies were included and was not downgraded for GS because the Pustejovsky–Rodgers test was not statistically significant (*p* = 0.6594). “Serious” and “very serious” indicate downgrading by one and two levels, respectively. “Not serious” indicates that no downgrading was applied for the corresponding GRADE domain. “Very low” indicates very limited confidence in the effect estimate, and the true effect may differ substantially from the estimated effect.

## Data Availability

The data supporting the findings of this study are available within the article and its Supplementary Materials.

## Supplementary Materials

The following supporting information can be downloaded at: https://www.mdpi.com/article/10.3390/bioengineering13080892/s1, Supplementary S1: Search strategies; Table S1: Training protocols of the included studies; Table S2: Classification and technical characteristics of the included robotic systems; Table S3: Randomized and outcome-specific analyzed sample sizes of the included studies; Figure S1: Leave-one-out sensitivity analyses; Figure S2: Device-class sensitivity analyses. Supplementary Tables S1–S3 and Figures S1 and S2 contain information derived from the included studies [9,10,11,21,22,23,24,25,26,27,28].

## Abbreviations

The following abbreviations are used in this manuscript:

RCT	randomized controlled trial
PRISMA	Preferred Reporting Items for Systematic Reviews and Meta-Analyses
PICOS	population, intervention, comparator, outcomes, and study design
SGA	spatial gait asymmetry
TGA	temporal gait asymmetry
GS	gait speed
CRT	conventional rehabilitation therapy
SMD	standardized mean difference
CI	confidence interval
REML	restricted maximum likelihood
RoB 2	Cochrane risk-of-bias tool for randomized trials, version 2
GRADE	Grading of Recommendations Assessment, Development and Evaluation
I^2^	heterogeneity statistic
τ^2^	between-study variance
