# Supplementary material for: Effects of Gait Training with Lower-Limb Robotic Exoskeletons and Exoskeleton-Type Devices on Gait Symmetry and Gait Speed in Patients with Stroke: A Systematic Review and Meta-Analysis
Source: Bioengineering (Basel). 2026 Aug 2;13(8):892. doi: 10.3390/bioengineering13080892 (PMC13509587; doi:10.3390/bioengineering13080892)
Supplement: Supplementary file 1 [file bioengineering-13-00892-s001.zip › bioengineering-4451687-supplementary.pdf]

Supplementary S1. Search strategies

June 21, 2026	Pubmed (530)
#1	("Stroke"[Mesh]OR stroke*[tiab]OR poststroke[tiab]OR post-stroke[tiab] OR (cerebrovascular[tiab] AND accident*[tiab])OR hemipleg*[tiab]OR hemipar*[tiab])
#2	("Exoskeleton Device"[Mesh]OR "Robotics"[Mesh]OR exoskeleton*[tiab]OR exosuit*[tiab]OR (wearable[tiab] AND robot*[tiab])OR (robot*[tiab] AND assist*[tiab] AND gait[tiab])OR (robot*[tiab] AND assist*[tiab] AND walk*[tiab])OR (robot*[tiab] AND lower[tiab] AND limb*[tiab])OR (powered[tiab] AND exoskeleton*[tiab])OR (soft[tiab] AND exosuit*[tiab])OR GEMS[tiab]OR EXOWALK[tiab]OR Kickstart[tiab]OR Healbot[tiab]OR SUBAR[tiab]OR (BEAR[tiab] AND H1[tiab])OR LiteStepper[tiab]OR curara[tiab]OR Welwalk[tiab])
#3	("Gait"[Mesh]OR "Walking"[Mesh]OR gait[tiab]OR walk*[tiab]OR locomot*[tiab]OR (lower[tiab] AND limb*[tiab])OR (lower[tiab] AND extremit*[tiab])OR hip[tiab]OR knee[tiab]OR ankle[tiab])
#4	(randomized controlled trial[pt]OR controlled clinical trial[pt]OR random*[tiab]OR trial[tiab]OR groups[tiab])
#5	#1 AND #2 AND #3 AND #4

June 21, 2026	Embase (721)
#1	'cerebrovascular accident'/exp OR 'hemiplegia'/exp OR 'hemiparesis'/exp OR (stroke* OR poststroke OR 'post stroke' OR 'post-stroke' OR 'cerebrovascular accident' OR 'cerebrovascular accidents' OR hemipleg* OR hemipar*):ti,ab,kw
#2	'exoskeleton'/exp OR 'robotics'/exp OR (exoskeleton* OR exosuit* OR 'robotic exoskeleton' OR 'powered exoskeleton' OR 'wearable robot' OR 'wearable robotic device' OR 'robot assisted gait training' OR 'robot-assisted gait training' OR 'robotic assisted gait training' OR 'robotic-assisted gait training' OR 'robotic gait training' OR 'robot assisted walking' OR 'robot-assisted walking' OR 'gait assist robot' OR 'walking assist robot' OR 'lower limb robot' OR 'lower extremity robot' OR 'soft robotic exosuit' OR GEMS OR EXOWALK OR Kickstart OR Healbot OR SUBAR OR LiteStepper OR curara OR Welwalk OR ReWalk OR Ekso OR Indego OR HAL OR 'BEAR H1'):ti,ab,kw
#3	'gait'/exp OR 'walking'/exp OR 'lower limb'/exp OR (gait OR walking OR walk* OR

	locomot* OR 'gait training' OR 'walking training' OR 'gait rehabilitation' OR 'gait symmetry' OR 'gait asymmetry' OR 'spatial symmetry' OR 'spatial asymmetry' OR 'temporal symmetry' OR 'temporal asymmetry' OR 'step length symmetry' OR 'step length asymmetry' OR 'swing time symmetry' OR 'swing time asymmetry' OR 'gait speed' OR 'walking speed' OR 'lower limb' OR 'lower limbs' OR 'lower extremity' OR 'lower extremities'):ti,ab,kw
#4	'randomized controlled trial'/exp OR 'controlled clinical trial'/exp OR 'randomization'/exp OR (random* OR randomized OR randomised OR randomly OR rct OR trial OR trials OR 'controlled trial' OR 'controlled trials' OR 'controlled study' OR 'controlled studies'):ti,ab,kw
#5	#1 AND #2 AND #3 AND #4

**June 21, 2026**	**Web of Science (376)**
#1	stroke* OR poststroke OR "post stroke" OR "post-stroke" OR "cerebrovascular accident" OR "cerebrovascular accidents" OR hemipleg* OR hemipar*
#2	exoskeleton* OR exosuit* OR "robotic exoskeleton" OR "robotic exoskeletons" OR "powered exoskeleton" OR "powered exoskeletons" OR "wearable robot" OR "wearable robots" OR "wearable robotic device" OR "wearable robotic devices" OR "robot assisted gait training" OR "robot-assisted gait training" OR "robotic assisted gait training" OR "robotic-assisted gait training" OR "robotic gait training" OR "robot assisted walking" OR "robot-assisted walking" OR "gait assist robot" OR "walking assist robot" OR "lower limb robot" OR "lower extremity robot" OR "soft robotic exosuit" OR "soft robotic exoskeleton" OR GEMS OR EXOWALK OR Kickstart OR Healbot OR "Healbot G" OR "Healbot-G" OR SUBAR OR LiteStepper OR curara OR Welwalk OR ReWalk OR Ekso OR Indego OR "BEAR H1" OR "BEAR-H1" OR "hybrid assistive limb"
#3	gait OR walking OR walk* OR locomot* OR "gait training" OR "walking training" OR "gait rehabilitation" OR "gait symmetry" OR "gait asymmetry" OR "spatial symmetry" OR "spatial asymmetry" OR "temporal symmetry" OR "temporal asymmetry" OR "step length symmetry" OR "step length asymmetry" OR "swing time symmetry" OR "swing time asymmetry" OR "gait speed" OR "walking speed" OR "lower limb" OR "lower limbs" OR "lower extremity" OR "lower extremities"

#4	random* OR RCT OR "randomized controlled trial" OR "randomized controlled trials" OR "randomised controlled trial" OR "randomised controlled trials" OR "controlled clinical trial" OR "controlled clinical trials" OR "controlled trial" OR "controlled trials" OR "clinical trial" OR "clinical trials"
#5	#1 AND #2 AND #3 AND #4

**June 21, 2026**	**Scopus (342)**
#1	TITLE-ABS-KEY(stroke* OR poststroke OR "post stroke" OR "post-stroke" OR "cerebrovascular accident" OR "cerebrovascular accidents" OR hemipleg* OR hemipar*)
#2	TITLE-ABS-KEY(exoskeleton* OR exosuit* OR "robotic exoskeleton" OR "robotic exoskeletons" OR "powered exoskeleton" OR "powered exoskeletons" OR "wearable robot" OR "wearable robots" OR "wearable robotic device" OR "wearable robotic devices" OR "robot assisted gait training" OR "robot-assisted gait training" OR "robotic assisted gait training" OR "robotic-assisted gait training" OR "robotic gait training" OR "robot assisted walking" OR "robot-assisted walking" OR "gait assist robot" OR "walking assist robot" OR "lower limb robot" OR "lower extremity robot" OR "soft robotic exosuit" OR "soft robotic exoskeleton" OR GEMS OR EXOWALK OR Kickstart OR Healbot OR "Healbot G" OR "Healbot-G" OR SUBAR OR LiteStepper OR curara OR Welwalk OR ReWalk OR Ekso OR Indego OR "BEAR H1" OR "BEAR-H1" OR "hybrid assistive limb")
#3	TITLE-ABS-KEY(random* OR RCT OR "randomized controlled trial" OR "randomized controlled trials" OR "randomised controlled trial" OR "randomised controlled trials" OR "controlled clinical trial" OR "controlled clinical trials" OR "controlled trial" OR "controlled trials" OR placebo* OR blind* OR crossover OR "cross over" OR "parallel group" OR "parallel groups")
#4	TITLE-ABS-KEY(randomized OR randomised OR randomly OR RCT)
#5	#1 AND #2 AND #3 AND #4

**June 21, 2026**	**Cochrane Library(336)**

#1	stroke* OR poststroke OR "post stroke" OR "post-stroke" OR "cerebrovascular accident" OR "cerebrovascular accidents" OR hemipleg* OR hemipar*
#2	exoskeleton* OR exosuit* OR "robotic exoskeleton" OR "powered exoskeleton" OR "wearable robot" OR "wearable robotic device" OR "robot assisted gait training" OR "robot-assisted gait training" OR "robotic assisted gait training" OR "robotic-assisted gait training" OR "robotic gait training" OR "robot assisted walking" OR "robot-assisted walking" OR "gait assist robot" OR "walking assist robot" OR "soft robotic exosuit" OR "soft robotic exoskeleton" OR GEMS OR EXOWALK OR Kickstart OR Healbot OR SUBAR OR LiteStepper OR curara OR Welwalk OR ReWalk OR Ekso OR Indego OR "BEAR H1" OR "hybrid assistive limb"
#3	gait OR walking OR walk* OR locomot* OR "gait training" OR "walking training" OR "gait rehabilitation" OR "gait symmetry" OR "gait asymmetry" OR "spatial symmetry" OR "spatial asymmetry" OR "temporal symmetry" OR "temporal asymmetry" OR "step length symmetry" OR "step length asymmetry" OR "swing time symmetry" OR "swing time asymmetry" OR "gait speed" OR "walking speed" OR "lower limb" OR "lower limbs" OR "lower extremity" OR "lower extremities"
#4	randomized OR randomised OR randomly OR RCT

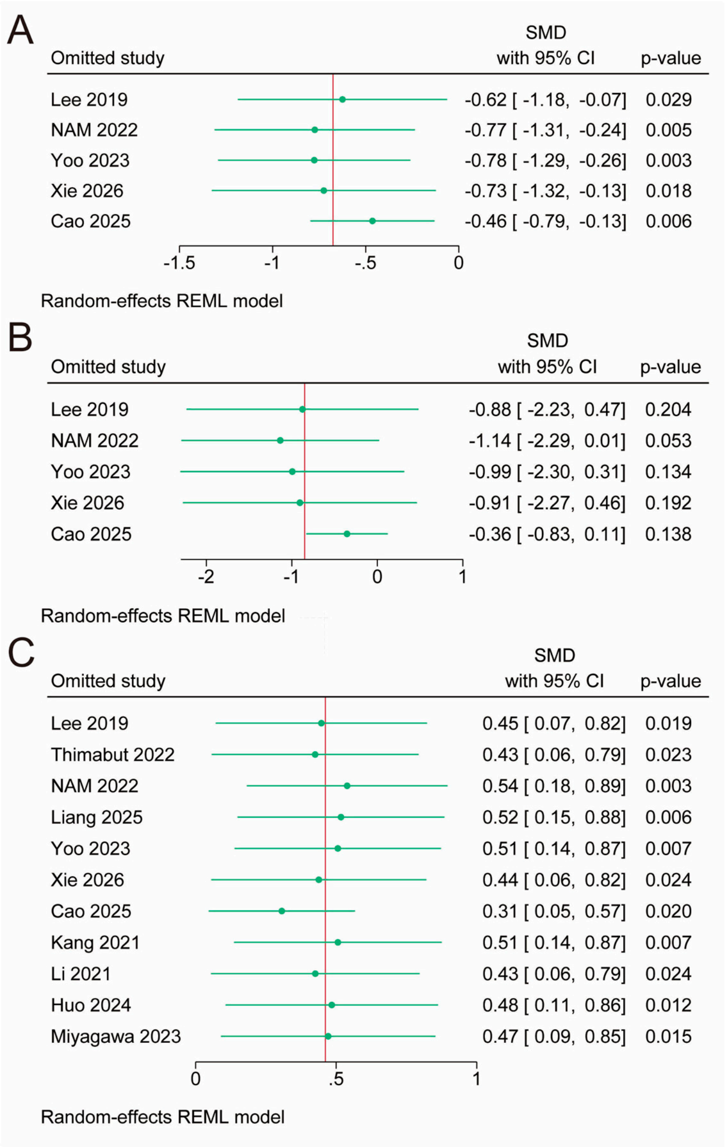

Supplementary Figure S1. Leave-one-out sensitivity analyses. A: spatial gait asymmetry (SGA) [9,23,24,26,27]; B: temporal gait asymmetry (TGA) [9,23,24,26,27]; C: gait speed (GS) [9–11,18,19,23–28]. Pooled analyses were performed using a random-effects REML model. SMD, standardized mean difference; CI, confidence interval.

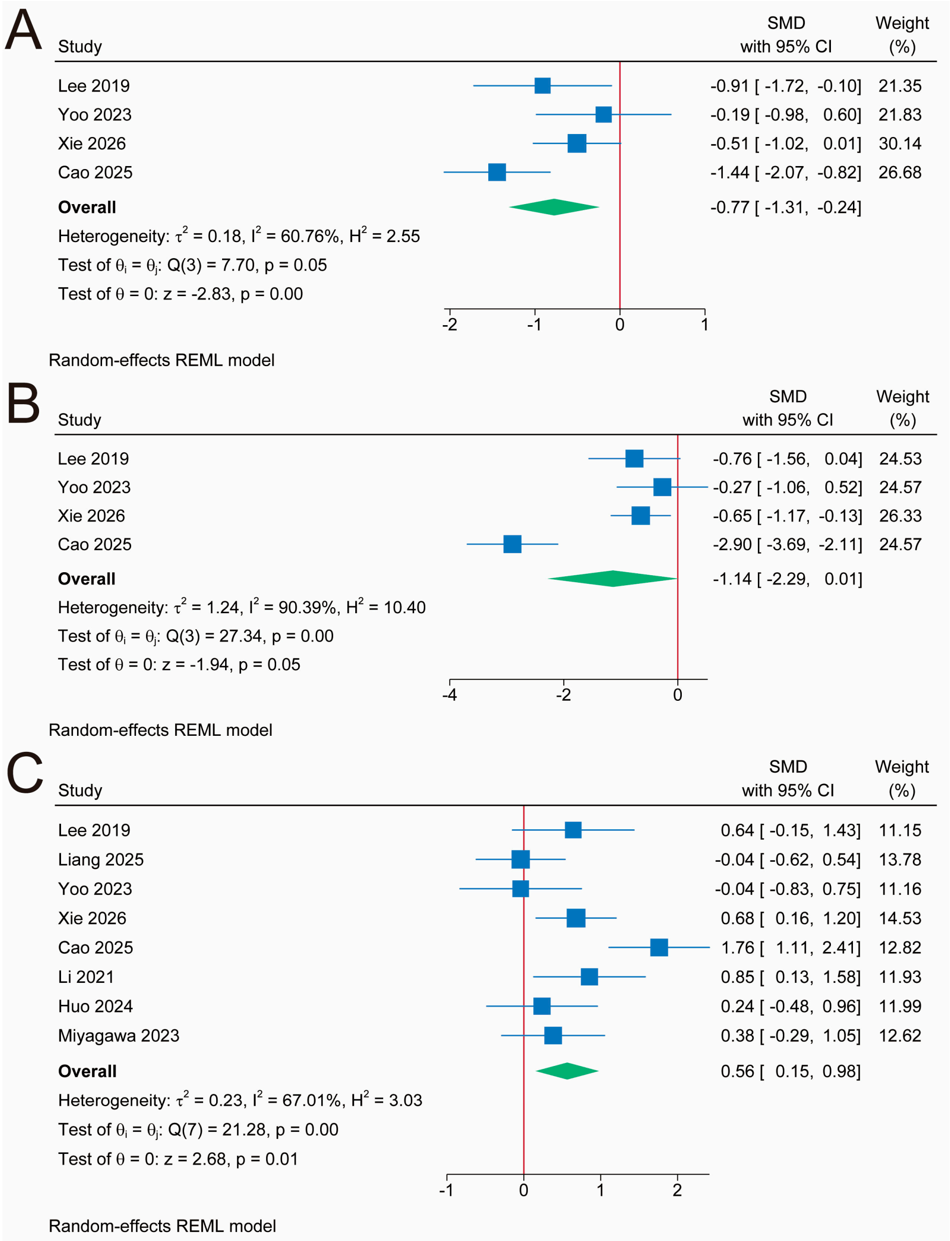

Supplementary Figure S2. Device-class sensitivity analyses. A: spatial gait asymmetry (SGA) after excluding the EXOWALK trial [9], based on studies [23,24,26,27]; B: temporal gait asymmetry (TGA) after excluding the EXOWALK trial [9], based on studies [23,24,26,27]; C: gait speed (GS) after excluding the Welwalk [18], EXOWALK [9], and SUBAR [19] trials, based on studies [10,11,23–28]. Pooled analyses were performed using a random-effects REML model. SMD, standardized mean difference; CI, confidence interval.

Supplementary Table S1. Training protocols of the included studies

Study	Device	Training mode/environment	Frequency	Session duration	Intervention duration	Total sessions	Concomitant rehabilitation	Control intervention
Lee et al. 2019 [23]	GEMS wearable bilateral hip-assist robot	Five treadmill and five overground sessions; treadmill training with a safety harness and no body-weight support; overground corridor training	3 sessions/week	45 min/session: 5-min warm-up, 35-min gait practice including a 5-min rest, and 5-min cool-down	4 weeks	10	NR	Identical treadmill and overground gait-training program without GEMS
Thimabut et al. 2022 [18]	Welwalk WW-1000 unilateral knee–ankle–foot robot with a low-floor treadmill and body-weight support	40-min robot-assisted treadmill training plus 20-min ground-level ambulation	1 session/day, 5 days/week	60-min ambulation training: 40-min robotic training plus 20-min ground-level training; additionally, 60-min conventional physiotherapy	6 weeks	30	Conventional physiotherapy, 60 min/session	Ground-level ambulation, 60 min/session, plus conventional physiotherapy, 60 min/session
Nam et al. 2022 [9]	EXOWALK electromechanical gait trainer	EXOWALK-assisted gait training at a comfortable speed of less than 1.8 km/h	5 sessions/week	30 min/session	4 weeks	20	Basic rehabilitation, including neurodevelopmental treatment, range-of-motion exercise, and strengthening; duration NR	Therapist-assisted conventional gait rehabilitation, 30 min/session, 5 sessions/week for 4 weeks, plus the same basic rehabilitation
Liang et al. 2025 [25]	Kickstart® Walk Assist passive wearable exoskeleton	Supervised overground gait training with Kickstart®	1 session/day, 5 days/week	20 min/session	4 weeks	20	Conventional rehabilitation, 40 min/day, 5 days/week for 4 weeks	Same conventional rehabilitation plus gait exercise training, 20 min/session, 5 days/week for 4 weeks
Yoo et al. 2023 [24]	Healbot G wearable powered exoskeleton	Treadmill gait training with Healbot G at a self-selected comfortable speed	NR; 10 sessions over 4 weeks	30 min/session	4 weeks	10	No additional rehabilitation described	Treadmill gait training without Healbot G, 30 min/session, for the same 10-session period
Xie et al. 2026 [26]	Yrobot Relink™ bilateral soft exoskeleton	Bilateral soft-exoskeleton-assisted treadmill walking	Once daily for 20 consecutive days	30 min/session	20 consecutive days	20	Conventional physical therapy, 30 min/day for 20 consecutive days	Same conventional physical therapy plus unassisted treadmill

								walking, 30 min/day for 20 consecutive days
Cao et al. 2025 [27]	Kickstart KS120-R01 passive wearable exoskeleton	Overground exoskeleton-assisted gait training combined with mirror-based posture feedback	1 session/day, 6 days/week	15 min/session	4 weeks	24	Comprehensive conventional hemiplegia rehabilitation, 30 min/day, 6 days/week for 4 weeks	Same conventional rehabilitation plus conventional assisted walking without a device, 15 min/day, 6 days/week for 4 weeks
Kang et al. 2021 [19]	SUBAR mobile overground lower-limb exoskeleton	Overground robot-assisted gait training; speed and step length were adjusted according to individual performance	NR; 10 sessions over 3 weeks	30 min/session	3 weeks	10	No additional rehabilitation described	Conventional physiotherapy based on neurodevelopmental treatment, 30 min/session, for 10 sessions over 3 weeks
Li et al. 2021 [28]	BEAR-H1 powered lower-limb exoskeleton	Overground BEAR-H1-assisted gait training using active and passive training modes	Twice daily, 5 days/week	30 min/session	4 weeks	40	Routine rehabilitation, including muscle strengthening, stretching, and balance training; duration NR	Conventional therapist-guided training, 30 min twice daily, 5 days/week for 4 weeks, plus the same routine rehabilitation
Huo et al. 2024 [10]	LiteStepper® unilateral powered lower-limb exoskeleton	Robot-assisted overground gait training	1 session/day, 5 days/week	30 min/session	4 weeks	20	Conventional rehabilitation, 5 days/week, including passive joint activity, strengthening, stretching, active control, transfer, balance, and walking training; duration NR	Conventional lower-limb walking rehabilitation, 30 min twice daily, 5 days/week for 4 weeks
Miyagawa et al. 2023 [11]	curara® type 4 wearable powered	Wearable robot-assisted gait training with individualized synchronization gain, gait cycle, and joint-angle settings	5 sessions/week	30 ± 5 min/session	15-day trial period	10	Physical therapy excluding gait training, occupational therapy, and/or speech therapy; total rehabilitation including gait training ≤3 h/day	Conventional therapist-assisted gait training, 30 ± 5 min/session, 5 sessions/week for 10 sessions, plus the same additional rehabilitation

Notes: NR, not reported. Total sessions refer to the scheduled gait-training sessions in the intervention group. For Yoo et al. (2023), the abstract states "10 times per week," whereas the Methods section states "10 sessions in total over 4 weeks"; the table follows the Methods section and records weekly frequency as NR. For Xie et al. (2026), the original report specifies 20 consecutive days. For Lee et al. (2019), the original study reported a 4-week intervention with three sessions per week and a total of 10 sessions, comprising five treadmill and five overground gait-training sessions; these values are presented as reported.

Supplementary Table S2. Classification and technical characteristics of the included robotic systems

Study	Device classification	Assisted side/joints	Mechanical/body-support configuration	Assistance/control strategy	Required patient effort	Therapist involvement	Classification rationale
Lee et al. 2019 [23]	Wearable powered hip-assist exoskeleton	Bilateral hip joints; flexion and extension	Lightweight body-worn unit (2.8 kg) with bilateral hip-mounted angular sensors and actuators. A safety harness was used during treadmill training without body-weight unloading.	Phase- and timing-based assistive torque and power were applied during hip flexion and extension. User weight and assistance level were set through a tablet interface.	Active walking was required at a self-selected or progressively increased speed; the device augmented rather than replaced voluntary gait.	A research administrator configured user weight and assistance level. Training was supervised, and the examiner progressed walking speed according to participant tolerance.	The actuators were worn directly on the pelvis/thigh region and provided joint-level assistance without dependence on a fixed or mobile external frame.
Thimabut et al. 2022 [18]	Platform-coupled exoskeleton-type system	Unilateral paretic limb; knee flexion/extension and swing advancement through a knee-ankle-foot robotic orthosis	A limb-mounted robotic orthosis was coupled to the Welwalk low-floor treadmill platform. Robot-weight cancellation was provided, and an overhead body-weight-support system could be used when trunk control was insufficient.	A plantar-pressure sensor identified gait-cycle events. Timed knee assistance and swing-out force were adjusted to provide the minimum support or guidance required; visual and auditory feedback were available.	Voluntary gait activity was encouraged, with robotic assistance adjusted to the patient's walking ability.	A trained therapist operated the control panel, adjusted knee-extension torque and swing assistance, selected feedback, and supervised safety.	Although coupled to a treadmill platform, a robotic orthotic component was attached directly to the paretic limb and delivered joint-level assistance; it was therefore distinct from an end-effector or treadmill-only system.
Nam et al. 2022 [9]	Mobile-frame exoskeleton-type gait trainer	Lower limbs; specific assisted joints were not reported	An exoskeletal structure was mounted in front of a mobile robotic body. The system provided stable upright support and moved on motorized wheels; no harness, cane, or walker was required.	The mobile robotic body was controlled by the patient and delivered repetitive training with a symmetrical gait pattern. Training speed was kept below 1.8 km/h.	The patient actively controlled progression of the mobile system and participated in walking; participants had to be able to stand independently.	Training was delivered by experienced physiotherapists. Detailed device-adjustment procedures and manual assistance were not reported.	The exoskeletal component was integrated with a mobile robotic frame rather than functioning as an independently wearable exoskeleton.
Liang et al. 2025 [25]	Wearable passive lower-limb exoskeleton	Unilateral affected limb; hip, knee, and ankle-foot	A belt, external support structure, Exotendon, universal hip joint, anti-hyperextension knee component, and ankle-foot orthosis were worn on the affected limb. No powered actuator or external body-weight-support frame was used.	The Exotendon stored energy during stance and released it during swing. Tendon tension was adjusted to assist hip flexion, limb advancement, toe clearance, and completion of the step.	Active overground walking was required; the passive device augmented the patient's own movement.	Gait training was supervised. A therapist fitted the device and adjusted tendon tension; detailed manual assistance during training was not reported.	The passive multijoint support was worn directly on the affected limb and provided mechanical assistance during gait.
Yoo et al. 2023 [24]	Wearable powered	Bilateral hip and knee joints; passive	A body-worn powered exoskeleton with four actuated hip-knee joints	Hip and knee joint angles and angular velocities were sensed,	Participants actively walked on the treadmill at a	Participants were monitored for safety and adverse events. Detailed	Powered actuators were worn directly on the lower limbs and

	lower-limb exoskeleton	loaded ankle support	and passive ankle components was used during treadmill walking. External body-weight support was not reported.	and a gait trajectory was generated using cubic-spline interpolation. Embedded control coordinated the four actuated joints.	self-selected comfortable speed while receiving powered joint guidance.	therapist control, parameter adjustment, or manual assistance was not reported.	controlled bilateral hip-knee motion without an external robotic frame.
Xie et al. 2026 [26]	Wearable bilateral soft exoskeleton	Bilateral ankle joints; plantarflexion and dorsiflexion	The Yrobot Relink system comprised actuators, Bowden cables, ankle braces, and rigid carbon-fiber ankle frames. An overhead safety system was present during treadmill walking, but string tension was set to zero.	IMU sensors detected heel strike and toe off, and force sensors monitored cable output. Gait-cycle-synchronized active plantarflexion assistance was delivered during late stance/push-off, while a torsion spring assisted dorsiflexion during swing.	Active treadmill walking was required; the device provided phase-specific bilateral ankle assistance.	Training was therapist-supervised. Therapists monitored tablet-based system parameters and individualized assistance levels, generally within 10-25% of body weight.	The cable-driven soft components and ankle braces were worn directly on both lower limbs and delivered joint-specific assistance.
Cao et al. 2025 [27]	Wearable passive lower-limb exoskeleton	Unilateral paretic limb; hip-knee-ankle linkage with facilitation of knee flexion	The passive Kickstart KS120-R01 was attached to the paretic limb through a belt, fixation components, moving components, and a pulley/linkage system connecting the hip, knee, and ankle. External body-weight support was not reported.	A ratchet mechanism adjusted linkage tension until a knee-flexion-assisting force was perceived. Mirror-based visual feedback was used for posture correction.	Participants walked voluntarily and used mirror feedback to actively correct gait posture.	Trained staff fitted the device. The therapist adjusted the device, assisted walking as needed, and guided correction of abnormal posture.	A passive multijoint mechanism was worn directly on the paretic limb and mechanically facilitated gait without an external robotic frame.
Kang et al. 2021 [19]	Mobile-frame overground exoskeleton-type system	Lower-limb hip, knee, and ankle movements; limb fastenings at thighs, calves, and feet	The SUBAR mobile frame supported body weight through buttock and anterior upper-trunk pedestals and guided the lower limbs through exoskeletal attachments. The system was not fixed to the floor and could move in hospital corridors.	Gait speed and step length were preset and progressed according to performance. A joystick controlled left-right direction of the mobile system.	Active lower-limb movement was supported; only participants with FAC >=3 were enrolled for safety.	Treatment was physiotherapist-supervised. Speed and step length were individualized, although detailed manual-assistance procedures were not reported.	The joint-guiding exoskeletal components were integrated with a large mobile body-support frame and were not independently wearable.
Li et al. 2021 [28]	Wearable powered bilateral	Bilateral hip, knee, and ankle joints; three active sagittal-	An adjustable rigid exoskeleton was aligned with the lower-limb joints	Brushless motors actuated the hip, knee, and ankle. Passive	Effort varied by mode: passive mode could guide	The operator measured limb length, fitted and secured the device, and set	The powered multijoint structure was worn directly on both lower

	lower-limb exoskeleton	plane degrees of freedom plus passive hip abduction/adduction in each leg	and fixed to the legs with straps. No external body-weight-support frame was described.	training, weight-loss, and intelligent active modes were available; the active mode adjusted stride frequency in real time to synchronize human-robot interaction.	gait, whereas intelligent active mode required and responded to voluntary participation.	parameters. Therapists or family members could monitor real-time gait data through the touch screen.	limbs and provided motorized joint assistance during overground gait.
Huo et al. 2024 [10]	Wearable unilateral powered lower-limb exoskeleton	Powered assistance to the paretic hip and knee, with coordinated assistance for hip, knee, and ankle movements; contralateral hip-knee sensing	A backpack-mounted host stabilized an affected-side powered exoskeleton and an unaffected-side sensing module. A pressure-sensing shoe and a trigger-equipped elbow crutch were also used; external body-weight support was not described.	Angle, torque, and plantar-pressure data from the unaffected side were used to generate synchronized targeted assistance for the paretic limb. A crutch-handle trigger initiated affected-side movement.	Active engagement of the unaffected limb was required; the paretic limb could be driven even when it generated little or no voluntary movement.	A physical therapist provided guidance and necessary assistance during gait training.	The powered modules were worn on the body and directly drove the paretic lower limb using contralateral gait information.
Miyagawa et al. 2023 [11]	Wearable powered non-rigid-link lower-limb robot	Bilateral hip and knee joints	The approximately 5-kg curara type 4 used four actuator units and a control box. Hip and knee actuator units had no direct rigid links and could function independently. External body-weight support was not reported.	Torque sensing and synchronization-based control were used. Synchronization gain, gait cycle, and joint-angle amplitudes were set, with greater assistance at the hip than at the knee.	Participants performed active overground gait while the device synchronized assistance with their movement and permitted intentional joint motion.	One or two physical therapists operated the device and prevented falls but did not provide gait advice.	The actuator units were worn directly at the hip and knee and provided synchronized joint assistance without a fixed platform.

Notes: Detailed training mode, frequency, session duration, intervention duration, total sessions, concomitant rehabilitation, and control interventions are reported in Supplementary Table S1 and are not repeated here. NR, not reported. Device classification was based on whether the joint-assisting component was independently wearable, integrated with a mobile body-support frame, or coupled to a treadmill/platform while remaining directly attached to the affected limb.

Supplementary Table S3. Randomized and outcome-specific analyzed sample sizes of the included studies

Study	Randomized sample, n	SGA analyzed sample, n	TGA analyzed sample, n	GS analyzed sample, n	Reason for the difference or unavailable data
Lee et al. 2019 [23]	28	26	26	26	Twenty-six participants completed the trial and were analyzed (14 intervention and 12 control). Two participants did not complete the trial; the reasons were not reported in the main article.
Thimabut et al. 2022 [18]	26	NR	NR	26	All randomized participants completed the study and were analyzed. The reported general gait-symmetry ratio was not classified into the prespecified SGA or TGA synthesis.

Nam et al. 2022 [9]	144	35	35	109	The post-intervention clinical walking analysis included 109 participants. Gait-symmetry analysis was limited to 35 participants who had usable instrumented gait data. Participants requiring walking aids or assistance and those for whom gait assessment was unsuitable or incomplete were not included in the symmetry analyses.
Liang et al. 2025 [25]	46	NR	NR	46	All randomized participants were analyzed for GS. Temporal symmetry ratio was reported as median and interquartile range and was not included because data suitable for the SMD calculation were unavailable.
Yoo et al. 2023 [24]	30	25	25	25	One participant did not receive the allocated intervention because of poor physical condition. Four additional participants were excluded from the per-protocol analysis because of failed data acquisition caused by motion artifacts or excessive noise.
Xie et al. 2026 [26]	60	60	60	60	One participant in each group did not complete the intervention because of early discharge; however, all 60 randomized participants were retained in the intention-to-treat analysis.
Cao et al. 2025 [27]	52	50	50	50	One participant in each group withdrew because treatment for another disease was required; 50 participants entered the final analysis.
Kang et al. 2021 [19]	30	NR	NR	30	All randomized participants were analyzed. Two participants in the robotic group completed only 9 of 10 sessions but remained in the analysis.
Li et al. 2021 [28]	36	NR	NR	32	Four participants withdrew for personal reasons: one from the intervention group and three from the control group.
Huo et al. 2024 [10]	40	NR	NR	30	Participants withdrew because of hospital discharge; two control-group participants were excluded because of disconnected gait data, and four robotic-group participants were excluded because of excessive artifacts. Thirty participants had complete analyzed data.
Miyagawa et al. 2023 [11]	40	NR	NR	35	Four participants discontinued the trial: two withdrew consent because of mental health problems, one developed a robot-related skin problem, and one experienced a recurrent stroke. One additional participant was excluded because of extreme outlying values.
Total	532	196	196	469	

Notes: SGA, spatial gait asymmetry; TGA, temporal gait asymmetry; GS, gait speed; SMD, standardized mean difference; NR, not reported or not included in the corresponding meta-analysis.

Section and Topic	Item #	Checklist item	Location where item is reported
**TITLE**			
Title	1	Identify the report as a systematic review.	page 1
**ABSTRACT**			
Abstract	2	See the PRISMA 2020 for Abstracts checklist.	page 1
**INTRODUCTION**			
Rationale	3	Describe the rationale for the review in the context of existing knowledge.	pages 2–3
Objectives	4	Provide an explicit statement of the objective(s) or question(s) the review addresses.	page 3

Section and Topic	Item #	Checklist item	Location where item is reported
**METHODS**			
Eligibility criteria	5	Specify the inclusion and exclusion criteria for the review and how studies were grouped for the syntheses.	pages 3–4
Information sources	6	Specify all databases, registers, websites, organisations, reference lists and other sources searched or consulted to identify studies. Specify the date when each source was last searched or consulted.	page 3
Search strategy	7	Present the full search strategies for all databases, registers and websites, including any filters and limits used.	page 3
Selection process	8	Specify the methods used to decide whether a study met the inclusion criteria of the review, including how many reviewers screened each record and each report retrieved, whether they worked independently, and if applicable, details of automation tools used in the process.	pages 4-5
Data collection process	9	Specify the methods used to collect data from reports, including how many reviewers collected data from each report, whether they worked independently, any processes for obtaining or confirming data from study investigators, and if applicable, details of automation tools used in the process.	pages 4–5
Data items	10a	List and define all outcomes for which data were sought. Specify whether all results that were compatible with each outcome domain in each study were sought (e.g. for all measures, time points, analyses), and if not, the methods used to decide which results to collect.	pages 5–6
	10b	List and define all other variables for which data were sought (e.g. participant and intervention characteristics, funding sources). Describe any assumptions made about any missing or unclear information.	pages 5–6
Study risk of bias assessment	11	Specify the methods used to assess risk of bias in the included studies, including details of the tool(s) used, how many reviewers assessed each study and whether they worked independently, and if applicable, details of automation tools used in the process.	pages 5
Effect measures	12	Specify for each outcome the effect measure(s) (e.g. risk ratio, mean difference) used in the synthesis or presentation of results.	page 5–6
Synthesis methods	13a	Describe the processes used to decide which studies were eligible for each synthesis (e.g. tabulating the study intervention characteristics and comparing against the planned groups for each synthesis (item #5)).	pages 5–6
	13b	Describe any methods required to prepare the data for presentation or synthesis, such as handling of missing summary statistics, or data conversions.	pages 5–6
	13c	Describe any methods used to tabulate or visually display results of individual studies and syntheses.	pages 5–6
	13d	Describe any methods used to synthesize results and provide a rationale for the choice(s). If meta-analysis was performed, describe the model(s), method(s) to identify the presence and extent of statistical heterogeneity, and software package(s) used.	pages 5–6
	13e	Describe any methods used to explore possible causes of heterogeneity among study results (e.g. subgroup analysis, meta-regression).	pages 5–6
	13f	Describe any sensitivity analyses conducted to assess robustness of the synthesized results.	pages 5–6

Section and Topic	Item #	Checklist item	Location where item is reported
Reporting bias assessment	14	Describe any methods used to assess risk of bias due to missing results in a synthesis (arising from reporting biases).	pages 5–6
Certainty assessment	15	Describe any methods used to assess certainty (or confidence) in the body of evidence for an outcome.	pages 5–6
**RESULTS**			
Study selection	16a	Describe the results of the search and selection process, from the number of records identified in the search to the number of studies included in the review, ideally using a flow diagram.	page 7
	16b	Cite studies that might appear to meet the inclusion criteria, but which were excluded, and explain why they were excluded.	page 7
Study characteristics	17	Cite each included study and present its characteristics.	pages 8–9
Risk of bias in studies	18	Present assessments of risk of bias for each included study.	pages 10–11
Results of individual studies	19	For all outcomes, present, for each study: (a) summary statistics for each group (where appropriate) and (b) an effect estimate and its precision (e.g. confidence/credible interval), ideally using structured tables or plots.	pages 11–13
Results of syntheses	20a	For each synthesis, briefly summarise the characteristics and risk of bias among contributing studies.	pages 11–13
	20b	Present results of all statistical syntheses conducted. If meta-analysis was done, present for each the summary estimate and its precision (e.g. confidence/credible interval) and measures of statistical heterogeneity. If comparing groups, describe the direction of the effect.	pages 11–13
	20c	Present results of all investigations of possible causes of heterogeneity among study results.	None
	20d	Present results of all sensitivity analyses conducted to assess the robustness of the synthesized results.	pages 11–13
Reporting biases	21	Present assessments of risk of bias due to missing results (arising from reporting biases) for each synthesis assessed.	page 13
Certainty of evidence	22	Present assessments of certainty (or confidence) in the body of evidence for each outcome assessed.	pages 14–15
**DISCUSSION**			
Discussion	23a	Provide a general interpretation of the results in the context of other evidence.	pages 15–16
	23b	Discuss any limitations of the evidence included in the review.	pages 18–19
	23c	Discuss any limitations of the review processes used.	pages 18–19

Section and Topic	Item #	Checklist item	Location where item is reported
	23d	Discuss implications of the results for practice, policy, and future research.	pages 18–19
**OTHER INFORMATION**			
Registration and protocol	24a	Provide registration information for the review, including register name and registration number, or state that the review was not registered.	page 3
	24b	Indicate where the review protocol can be accessed, or state that a protocol was not prepared.	page 3
	24c	Describe and explain any amendments to information provided at registration or in the protocol.	Not reported
Support	25	Describe sources of financial or non-financial support for the review, and the role of the funders or sponsors in the review.	None
Competing interests	26	Declare any competing interests of review authors.	page 20
Availability of data, code and other materials	27	Report which of the following are publicly available and where they can be found: template data collection forms; data extracted from included studies; data used for all analyses; analytic code; any other materials used in the review.	Supplementary Materials
